# Plasma Profiles of Neuroglial Injury Biomarkers after Ischemic Stroke

**DOI:** 10.1007/s12975-025-01380-y

**Published:** 2025-09-03

**Authors:** Karl Sjölin, Björn Röyter, Bianka Forgo, Julia Aulin, Kim Kultima, Johan Lindbäck, Jakob O. Ström, Joachim Burman

**Affiliations:** 1 Department of Medical Sciences, Translational Neurology, Uppsala University, Uppsala, Sweden; 2 Department of Obstetrics and Gynecology, Faculty of Medicine and Health, Örebro University, Örebro, Sweden; 3 Department of Neuroradiology, Karolinska University Hospital, Stockholm, Sweden; 4 Department of Clinical Neuroscience, Karolinska Institutet, Stockholm, Sweden; 5 Faculty of Medicine and Health, Örebro University, Örebro, Sweden; 6 Department of Medical Sciences, Cardiology, Uppsala University, Uppsala, Sweden; 7 Uppsala Clinical Research Center (UCR), Uppsala University, Uppsala, Sweden; 8 Department of Medical Sciences, Clinical Chemistry, Uppsala University, Uppsala, Sweden; 9 Department of Neurology, Faculty of Medicine and Health, Örebro University, Örebro, Sweden

**Keywords:** Stroke, Neurofilament, Glial fibrillary acidic protein, Tau, Ubiquitin carboxy-terminal hydrolase L1

## Abstract

****Objective**:**

To determine the temporal profiles of glial fibrillary acidic protein (GFAP), neurofilament light (NFL), total tau (t-tau), and ubiquitin carboxy-terminal hydrolase L1 (UCHL1) in plasma the first week after acute ischemic stroke, and identify the optimal time points for assessing infarct volume by these biomarkers.

****Patients & Methods**:**

In this cohort study, biomarker plasma concentrations were determined daily over the first week and at 90 days after symptom onset in patients with acute ischemic stroke. A brain MRI was performed on day three. Temporal variations in biomarker levels were analyzed using linear mixed-effects models, and optimal time points for infarct volume correlation were identified with continuous Pearson analysis.

****Results**:**

38 patients with a median age of 78 (IQR 72–86) and mean infarct volume of 5.5 (IQR 1.6–17) cm^3^ were included. We identified three distinct temporal patterns: (1) a parabolic trajectory of GFAP, reaching zenith after three days, (2) a consistent increase in NFL throughout the week, and (3) an initial surge in t-tau and UCHL1 levels, stabilizing by day three. The optimal time point for infarct volume correlation occurred at 119 h for GFAP (r = 0.94, 95% CI: [0.84–0.98]), 144 h for NFL (r = 0.78, [0.47, 0.92]), 122 h for t-tau (r = 0.82, [0.56, 0.93]) and 113 h for UCHL1 (r = 0.83, [0.60, 0.93]).

****Interpretation**:**

This high-resolution serial sampling of plasma GFAP, NFL, t-tau, and UCHL1 the first week after acute ischemic stroke identified three distinct temporal profiles. These biomarkers provided the most accurate infarct volume assessment 4–6 days after symptom onset. Clinicaltrials.gov NCT03812666 (registration date 2019-01-23).

**Supplementary Information:**

The online version contains supplementary material available at 10.1007/s12975-025-01380-y.

## Introduction

The management of acute stroke has evolved considerably over the past decades, leading to decreased mortality and lower risk of recurrence in high-income countries [1, 2]. The acute stroke diagnosis is based on typical medical history and clinical examination together with neuroimaging to differentiate between ischemic and hemorrhagic stroke. Although neuroimaging is the reference standard for assessing structural injury to the brain after an ischemic stroke, this does not imply that it accurately captures all pathological changes [3]. Molecular biomarkers could enhance the accuracy of estimating the extent of tissue injury as well as other pathological brain changes after a stroke. In contrast to the remarkable development in other areas of stroke care, molecular biomarkers to assist treatment decisions have not been implemented [4]. There are many applications and scenarios where biomarkers could be of significant importance, e.g., to differentiate between ischemic and hemorrhagic stroke, to assess the amount of viable versus non-viable tissue in the ischemic region, or to predict outcomes [4–6].

The neuroglial proteins glial fibrillary acidic protein (GFAP), neurofilament light (NFL), tau, and ubiquitin carboxy-terminal hydrolase L1 (UCHL1) are abundant in different cell types and anatomical regions in the central nervous system (CNS) [7]. These proteins have gained attention in the setting of stroke, as they are released into the blood after structural injury to the brain. Multiple studies have shown a correlation between absolute or change in plasma or serum levels of these proteins and infarct volume, as well as clinical outcomes in ischemic stroke [8–20]. However, most of these studies have relied upon a single blood sample or samples collected at a few time points. It is presently not known precisely when peak levels occur or when the correlation to infarct volume is optimal, which are key to interpreting individual levels correctly. Moreover, understanding biomarker dynamics and their relation with brain tissue injury provides pathophysiological insights and is important for comparing studies and validating biomarkers for clinical use.

The aims of this study were to describe the temporal profiles of GFAP, NFL, total tau (t-tau), and UCHL1 in plasma during the first week after acute ischemic stroke and determine the best time point to assess infarct volume with these biomarkers.

## Material and Methods

### Ethical Approval & Informed Consent

This study was performed in accordance with the Helsinki Declaration and approved by the regional ethical review board in Uppsala, Sweden (Dnr: 2018/288) and the Swedish Ethical Review Authority (Dnr: 2020–06000). In cases where patients were unable to provide informed consent due to aphasia or other cognitive disturbances, consent was obtained from next of kin. All other patients provided written informed consent.

### Study Design, Participants, and Data Collection

This observational study (Clinicaltrials.gov ID# NCT03812666) prospectively included patients with acute ischemic stroke admitted to the stroke unit at Örebro University Hospital between 2019 and 2021. Inclusion criteria were symptoms and radiological findings consistent with acute cerebral infarction and symptom onset less than 48 h before the first study blood sample collection. Patients with hemorrhagic stroke or radiological evidence of acute cerebral infarction but complete reversal of symptoms within 24 h were excluded (i.e., transient ischemic attack [TIA]). Other exclusion criteria were contraindications to magnetic resonance imaging (MRI) and ongoing inflammatory disease. Patients fulfilling inclusion and exclusion criteria within the time frame were consecutively asked to participate in the study. Baseline clinical data included age, sex, time of symptom onset, pre-stroke disability using the modified Rankin scale (mRS), and stroke severity using the National Institutes of Health Stroke Scale (NIHSS). The modified Rankin scale ranges from 0 to 6, with higher scores indicating more significant disability [21]. NIHSS ranges from 0 to 42, with higher scores indicating worse neurological deficits [22]. Comorbidities, renal function, and reperfusion treatment were assessed by reviewing medical records. NIHSS and mRS were evaluated by a study nurse at a follow-up visit after 90 days.

### Neuroimaging

Patients underwent MRI between 48–72 h after hospital admission on a Philips Achieva dStream 1.5 Tesla scanner (Philips, Best, The Netherlands). The protocol included two-dimensional T2-weighted axial and diffusion-weighted images (DWI). T2-weighted images were acquired using the following parameters: repetition time 4000 ms, echo time 110 ms, slice thickness 3 mm, in-plane resolution 0.4 × 0.4 mm. DWI was obtained with b-value 1000 s/mm^2^, repetition time 3704 ms, echo time 92 ms, slice thickness 5 mm, and 1.3 × 1.3 mm in-plane resolution.

Total volumes of ischemic infarcts were assessed on imaging. All measurements were made by a board-certified radiologist and neuroradiology trainee (BF). Infarct volumes were quantified on DWI using MRIcron (https://people.cas.sc.edu/rorden/mricron/index.html) with semi-automatic segmentation and manual correction if needed. Apparent diffusion coefficient maps and T2-weighted images were used to delineate acute infarcts.

### Biochemical Analyses

Venous blood samples were collected daily during the first week after stroke onset and at the 90-day follow-up visit. Blood samples were aliquoted and stored at −80 °C. The concentrations of GFAP, NFL, t-tau, and UCHL1 were determined in plasma with Single Molecule Array (Simoa®) using the Neurology 4-Plex B assay on the SR-X™ Biomarker Detection System (Quanterix Corp., MA, USA). The lower limit of quantification and the lower limit of detection was 9.38 pg/ml and 1.04 pg/ml for GFAP, 0.625 pg/ml and 0.0989 pg/ml for NFL, 0.100 pg/ml and 0.0281 pg/ml for t-tau, and 9.38 pg/ml and 1.13 pg/ml for UCHL1. The analyses were run in batches by the same board-certified technician at the Department of Clinical Chemistry, Uppsala University Hospital, Sweden.

### Statistical Analyses

The modeling of the kinetics of the biomarkers was based on the assumption that the plasma concentrations were dependent on both time from symptom onset and infarct volume. First, a linear mixed-effects model was used to assess the variation in biomarker concentration over time:

$$log(C) \sim rcs(T) + (1 + T | id)$$

where C is the biomarker concentration, rcs(T) is a restricted cubic spline function of time as a fixed effect, and (1 + T | id) represents the random effects (intercept and slope) for subjects. This means that the functional form of the average time trend was assumed the same for all patients. Still, the intercept (overall concentration level of the curve) and the (linear) slope of the curves were allowed to vary between patients.

After that, the effect of total infarct volume, V, on biomarker concentrations was assessed by adding log(V) to the model:

$$log(C) \sim log(V) + rcs(T) + (1 + T | id)$$

When plotting the volume-adjusted concentration curves, the regression coefficient for log(V) was used to adjust for infarct volume for each individual. The log(V) regression coefficient was close to 1/3 for all biomarkers except for GFAP, where it was close to 2/3, leading to units of pg/ml/cm for NFL, t-tau, and UCHL1 and ng/ml/cm^2^ for GFAP, after back transformation. The effect of accounting for volume is reported as the change in interindividual variance (square of standard deviation) of the log concentration.

Adjustments for age and sex were done by fitting a linear mixed-effects model with the volume-normalized biomarker level as outcome, accounting for the correlation between measurements within the same subject. Time since onset, age and sex were included as fixed effects. The estimated average volume-normalized biomarker level over time was plotted using predictions from the model for both sexes and three arbitrarily selected age groups (Fig. S1-S4, Supplementary Material).

To assess the time point with the highest correlation to infarct volume (T_optimal_), a sequential analysis was done by calculating the Pearson correlation between biomarker level and the single time point infarct volume measurement by MRI, for all biomarker measurements within a moving window of ± 12 h at each plotted hour. This is presented graphically as a moving 24 h correlation. A statement of significance implies statistically significant at the 5% significance level from two-sided tests.

## Results

### Clinical Characteristics

In total, 38 patients were included in the study (Table 1). Inclusion was temporarily suspended during specific periods of the COVID-19 pandemic. All patients received a final diagnosis of acute ischemic stroke. Median age was 78 years (interquartile range, IQR 72–86), and 47% were female. A third of the patients (29%) had a history of stroke or TIA. Hypertension (61%), atrial fibrillation (39%), and a diagnosis of hyperlipidemia (24%) were common. Median NIHSS at inclusion was 5 (IQR 2–10), and median infarct volume was 5.5 (IQR 1.6–17) cm^3^. Five patients did not undergo MRI due to death (*n* = 2), unknown (*n* = 2), and patient declined (*n* = 1), and were therefore excluded in the infarct adjusted analysis. At the 90-day follow-up, median NIHSS was 2 (IQR 1–4, *n* = 21), and median mRS was 1 (IQR 1–4, *n* = 27). All patients provided blood samples at a median of 5 (IQR 4–7) different time points, with the first blood sample collected at a median of 31 (IQR 23–40) hours after symptom onset. In total, 205 blood samples were analyzed.

**Table 1:** Clinical characteristics. Binary variables are presented as number (percent), all other variables are presented as median (interquartile range). eGFR, estimated glomerular filtration rate; IQR, interquartile range; mRS, modified Rankin scale; NIHSS, National Institutes of Health Stroke Scale; TIA, transient ischemic attack

Patients, no	*N* = 38
Age, median (IQR), yrs	78 (72–86)
Female sex, no. (%)	18 (47)
Previous stroke or TIA, no. (%)	11 (29)
Hypertension, no. (%)	23 (61)
Atrial fibrillation, no. (%)	15 (39)
Diabetes, no. (%)	5 (13)
Hyperlipidemia, no. (%)	9 (24)
Coronary artery disease, no. (%)	3 (7.9)
Heart failure, no. (%)	2 (5.3)
NIHSS at inclusion, median (IQR)	5 (2–10)
Pre-stroke mRS, median (IQR)	1 (0–3)
Infarct volume, median (IQR), cm^3^ (n = 33)	5.5 (1.6–17)
eGFR, median (IQR), ml/min per 1.73 m^2^	67 (54–79)
Treatment with intravenous thrombolysis, no. (%)	5 (13%)
90 days NIHSS, median (IQR) (n = 21)	2 (1–4)
90 days mRS, median (IQR) (n = 27)	1 (1–4)

### Temporal Profile

**Fig. 1:**
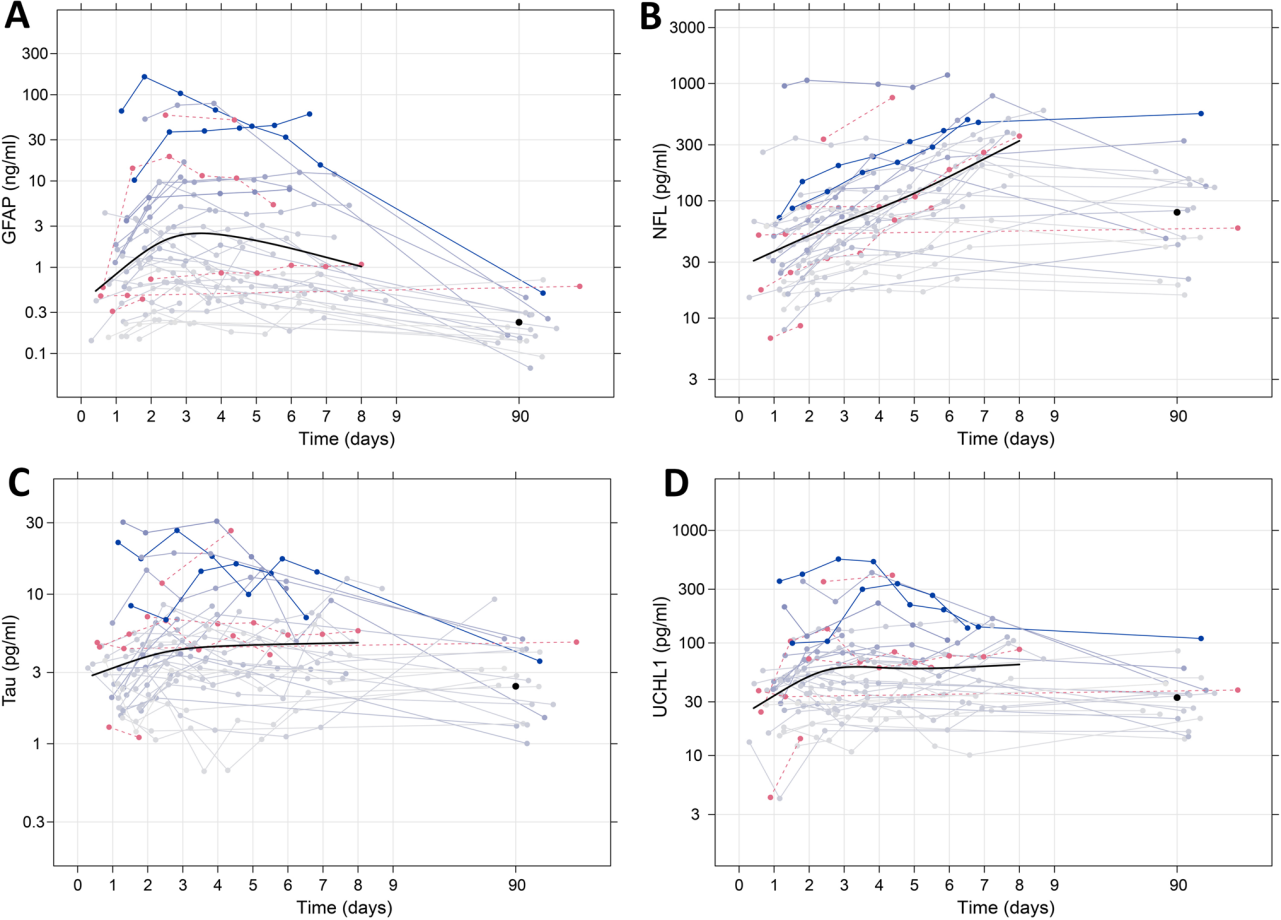
Temporal profile of neuroglial injury biomarkers. Plasma concentrations of GFAP (**A**), NFL (**B**), tau (**C**), and UCHL1 (**D**) the first week after ischemic stroke. Each solid blue line represents a single patient, with darker blue indicating patients with larger infarct volumes. Patients who did not undergo an MRI are indicated with red dashed lines.. The solid smooth black line indicates a restricted cubic spline fitted to all observations up to 9 days, and the solid black dot is the mean at 90 days. Deviations of times around 90 days have been scaled by a factor of 1/10 such that 10 days are related to 90 days as much as 1 day is related to 9 days in the rest of the plot. *N* = 38. GFAP, glial fibrillary acidic protein; NFL, neurofilament light; UCHL1, ubiquitin carboxy-terminal hydrolase L1

**Fig. 2:**
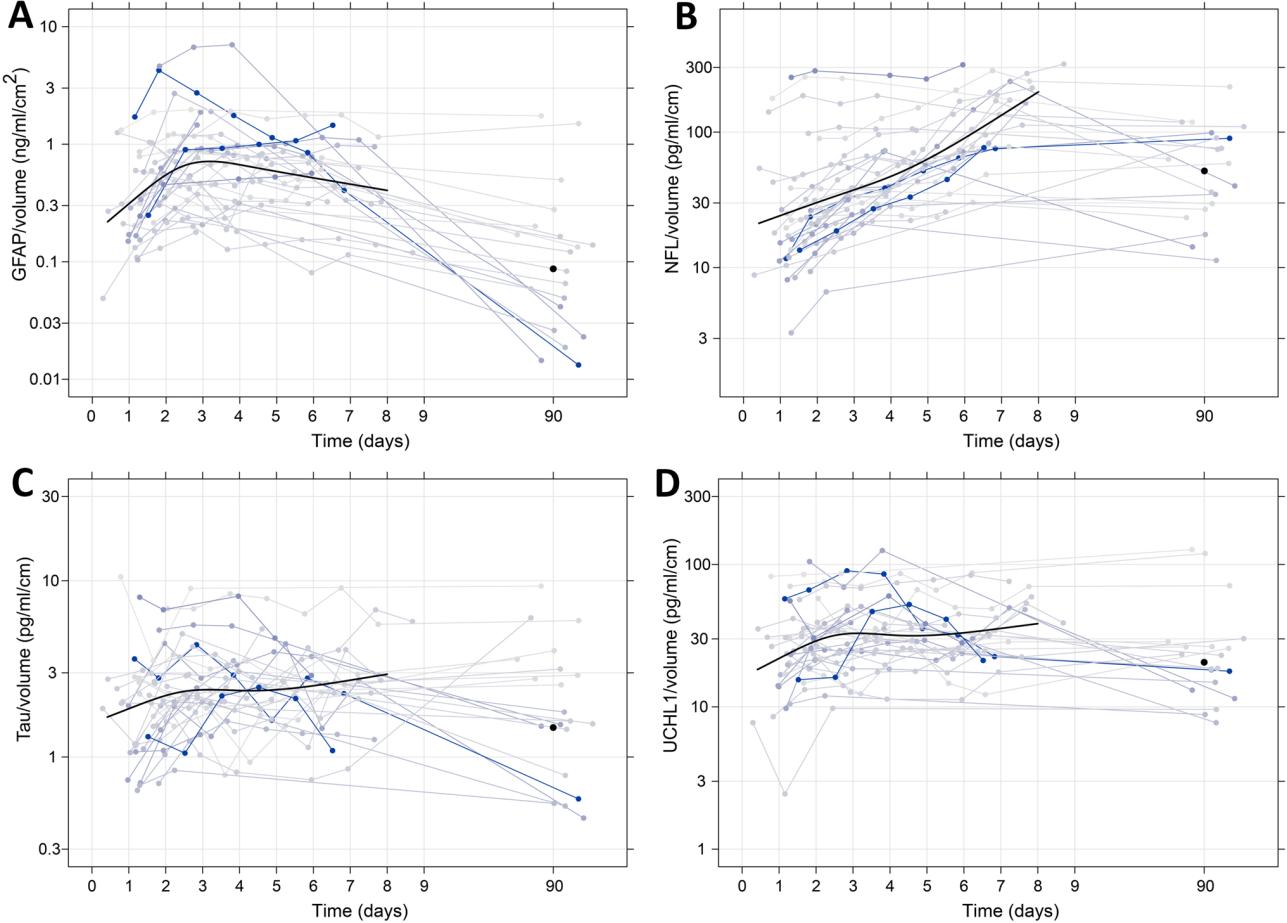
Temporal profile of neuroglial injury biomarkers normalized to infarct volume. Plasma concentrations of GFAP (A), NFL (**B**), tau (**C**), and UCHL1 (**D**) after ischemic stroke normalized to infarct volume. Each solid blue line represents a single patient, with darker blue indicating patients with larger infarct volumes. The solid smooth black line indicates a restricted cubic spline fitted to all observations up to 9 days, and the solid black dot is the mean at 90 days. Deviations of times around 90 days have been scaled by a factor of 1/10 such that 10 days are related to 90 days as much as 1 day is related to 9 days in the rest of the plot. *N* = 33. GFAP, glial fibrillary acidic protein; NFL, neurofilament light; UCHL1, ubiquitin carboxy-terminal hydrolase L1

Unadjusted concentrations during the first week and after 90 days for all patients and all biomarkers are presented in Fig. 1. The peak level occurred on average at 82 h (3 days) for GFAP and > 8 days for NFL, t-tau, and UCHL1. After adjustment for infarct volume, peak level time points for GFAP and NFL were essentially unaltered, occurring at 77 h (3 days) for GFAP and > 8 days for NFL, t-tau, and UCHL1 after stroke onset (Fig. 2). Patients with larger infarct volume generally had higher levels of all biomarkers (as indicated by darker blue in Fig. 1). There were no substantial differences across sex and age groups, with the exception of slightly higher NFL levels observed in women compared to men (Fig. S1-S4).

Three distinct temporal profiles were observed. GFAP inclined steeply during the first 3 days after stroke onset, followed by a gradual decline on days 4–7. In contrast, NFL displayed a steady, almost linear (on a logarithmic scale) increase during the first week. T-tau and UCHL1 demonstrated an initial increase followed by a plateau by day 3, with a slight increase in plasma levels at the end of the week. At 90 days follow-up, the concentration of each neuroglial biomarker was lower than the concentration of the last measurement of the first week.

By adjusting for log volume, the interindividual variance (square of standard deviation) of the log concentration was reduced by 54% for GFAP, 34% for t-tau, and 40% for UCHL1. In contrast, it increased by 16% for NFL.

### Correlation with Infarct Volume

**Fig. 3:**
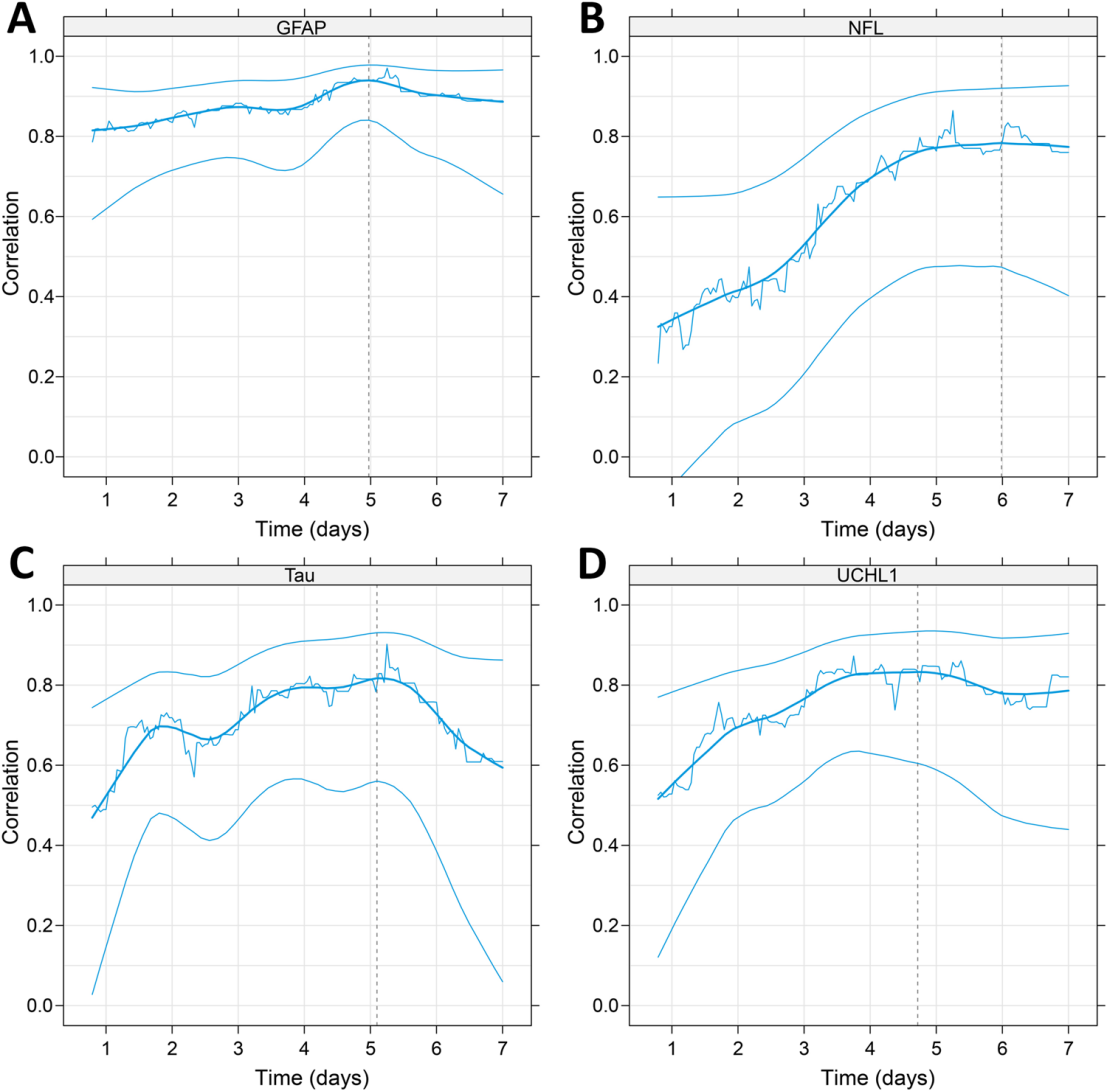
Continuous correlation with infarct volume. Continuous Pearson correlation between infarct volume and log concentration of GFAP (**A**), NFL (**B**), tau (**C**) and UCHL1 (**D**) over the first week. The correlation is calculated for all observations within a window of ± 12 h at each plotted hour. It can thus be interpreted as a moving 24-h correlation. The thick blue line shows a non-parametric smoother of the correlations. The thin blue lines are, correspondingly, smoothed 95% confidence limits for the correlation. The black vertical dotted lines indicate the time point for maximum correlation, T_optimal_, which occurred at 119 h (5.0 days) for GFAP (r = 0.94, 95% confidence interval, CI: [0.84–0.98]), 144 h (6.0 days) for NFL (r = 0.78, [0.47, 0.92]), 122 h (5.1 days) for t-tau (r = 0.82, [0.56, 0.93]), and at 113 h (4.7 days) for UCHL1 (r = 0.83, [0.60, 0.93]) N = 33. GFAP, glial fibrillary acidic protein; NFL, neurofilament light; UCHL1, ubiquitin carboxy-terminal hydrolase L1

**Fig. 4:**
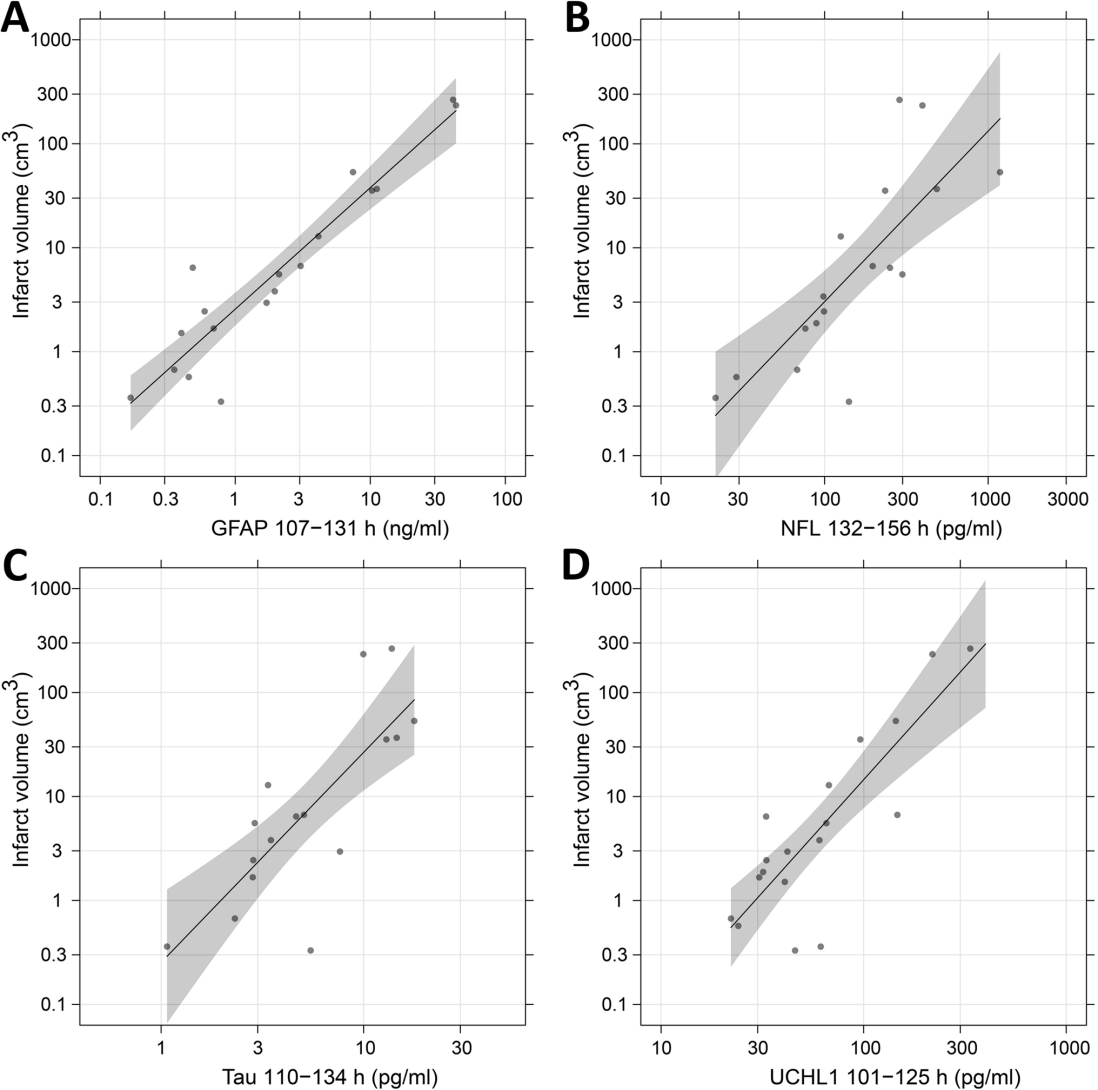
Correlation with infarct volume at optimal time point. Infarct volume versus plasma concentration of GFAP (**A**), NFL (**B**), tau (**C**) and UCHL1 (**D**) at the time of maximum correlation, T_optimal_ ± 12 h, which occurred at 119 h (5.0 days) for GFAP (r = 0.94, 95% CI: [0.84–0.98]), 144 h (6.0 days) for NFL (r = 0.78, [0.47, 0.92]), 122 h (5.1 days) for t-tau (r = 0.82, [0.56, 0.93]), and at 113 h (4.7 days) for UCHL1 (r = 0.83, [0.60, 0.93]). If a patient had more than one observation within the interval, the first observation was used. CI, confidence interval; GFAP, glial fibrillary acidic protein; NFL, neurofilament light; UCHL1, ubiquitin carboxy-terminal hydrolase L1

The correlation between biomarker level and infarct volume within a moving window of 24 h is presented in Fig. 3. The time point with highest correlation to infarct volume (T_optimal_) occurred at 119 h (5.0 days) for GFAP (r = 0.94, 95% confidence interval, CI: [0.84–0.98]), 144 h (6.0 days) for NFL (r = 0.78, [0.47, 0.92]), 122 h (5.1 days) for t-tau (r = 0.82, [0.56, 0.93]), and at 113 h (4.7 days) for UCHL1 (r = 0.83, [0.60, 0.93]) after symptom onset. At the 90-day follow-up, NFL and UCHL1 were significantly correlated with infarct volume (r = 0.55, *P* = 0.01 for NFL, and r = 0.57, *P* = 0.01 for UCHL1), whereas GFAP and t-tau were not (r = 0.43, *P* = 0.060 for GFAP, and r = 0.15, *P* = 0.53 for t-tau). Figure 4 shows the correlation between biomarker concentration and infarct volume at T_optimal_ within a window of ± 12 h. For comparison, the correlation between NIHSS at inclusion and log infarct volume was r = 0.47 (95% CI 0.16–0.70, *P* = 0.005).

## Discussion

In this study, we charted the temporal profile of GFAP, NFL, t-tau, and UCHL1 concentrations in plasma following an ischemic stroke by analyzing daily blood samples collected throughout the first week after symptom onset. This approach enabled the identification of peak biomarker levels and the optimal time point to assess infarct volume. GFAP exhibited a parabolic trajectory, reaching its zenith on the third day after the cerebral insult. NFL levels increased continuously, suggesting the peak extends beyond the weeklong observation window. Both t-tau and UCHL1 demonstrated an early peak on day three before stabilizing into a plateau. After adjustment for infarct volume, the inter-individual variation of GFAP, t-tau, and UCHL1 decreased. The correlation between biomarker level and infarct volume was generally stronger towards the end of the first week for all biomarkers, except GFAP, which correlated strongly with infarct volume already at 24 h. At 90 days, NFL and UCHL1 were the only biomarkers that remained significantly correlated to infarct volume.

The findings of this study are consistent with and expand on previous reports. In particular, the temporal profile of NFL in plasma or serum after stroke has been characterized over the first weeks and months after stroke [20, 23]. According to these reports, the peak of NFL occurs about two weeks after an ischemic stroke and could, therefore, not be captured in our study. The strength of the correlations between NFL levels and infarct volume increased over the initial five days and was optimal six days after stroke onset. Earlier research has documented an increasing strength of correlation between NFL levels and infarct size from day three to day seven post-stroke [8, 10, 12]. Notably, studies focusing on very early sampling time points (within the first 24 h) did not establish a significant association between NFL levels and infarct size, underscoring the importance of timing in biomarker analyses [11, 16, 24].

Less is documented regarding the plasma or serum profile of GFAP after ischemic stroke. In two recent studies with serial sampling the first three days after endovascular treatment (EVT) due to large vessel occlusion stroke, a peak of GFAP was noted two to three days after the procedure, with a serum profile similar to our findings, especially for patients with poor outcome (defined as increased, stabilized, or less than four points improvement from the initial score on NIHSS) [17, 18]. With a few exceptions, patients in our study displayed an increase in GFAP between the first and second samples, indicating that GFAP might be a robust early stroke biomarker. The early rate of change in GFAP levels has been reported to be highly correlated to outcome, as well as to discriminate between ischemic and hemorrhagic stroke [17, 25]. A prompt increase of serum GFAP was also observed in two earlier studies using a less sensitive ELISA, where a clear peak was discerned after two and four days respectively in patients with large infarcts [26, 27]. In our study, a strong correlation between GFAP levels and infarct volume was observed from day one and throughout the first week, with the highest correlation identified at day five. Similarly, and in contrast to NFL, others have also reported a significant correlation between GFAP and infarct volume at the early time points after stroke onset in patients with large vessel occlusion and patients undergoing EVT [16, 18]. Notably, GFAP was the only biomarker among several that correlated with infarct volume at the earliest time point two hours after EVT [18]. In summary, the correlation between GFAP and infarct volume appears to be less timing-dependent during the first week than the other investigated biomarkers in our study.

Some previous data exist regarding the temporal profile of t-tau after stroke, although most studies have focused on the first three days after onset or at follow-up visits made months after the cerebral insult [15, 17, 18, 25, 28]. However, with blood samples collected at four time points over the first week after stroke, one study reported a prominent increase in plasma t-tau over the first three days, with a slight rise at day seven after stroke onset, similar to our findings [29]. In earlier studies on t-tau in serum after stroke using standard ELISA, t-tau was below the detection limit in more than half of the patients studied [30–32]. Nevertheless, a rise of t-tau levels was discernable after two days in one of the studies that included only patients with middle cerebral artery occlusion [31]. This highlights the importance of high-sensitivity biochemical analysis when evaluating these proteins in plasma or serum after stroke. The correlation between t-tau and infarct volume became stronger during the first days up to the optimal time point five days after stroke onset. Considering previous reports, it seems likely that the optimal time point for correlating tau with infarct volume occurs between days four and seven during the first week [18, 29]. However, considerable within-subject variability across different time points was observed for t-tau. The underlying reasons for this, whether stemming from preanalytical or analytical factors, inherent biological processes, or the fact that tau is not exclusively expressed in the CNS, remain undetermined.

Few studies have investigated plasma or serum levels of UCHL1 after stroke. We found only one study examining UCHL1 beyond 48 h after stroke. In a randomized controlled trial investigating the effect of erythropoietin in acute ischemic stroke, a slight increase in UCHL1 during the first three days after stroke was noted in the placebo group but not in the treatment group; however, no imaging data was available [33]. Another study failed to show any difference in admission serum levels of UCHL1 between patients with ischemic stroke and a control group [34]. Of note is that in both these studies, standard ELISA was used, which may not have quantified UCHL1 correctly. In a study of biomarker levels after EVT, a slight increase of UCHL1 was observed 24 h after the procedure compared to before, with only a modest correlation to infarct volume [16]. In our study, the temporal profile of UCHL1 resembled that of t-tau and similarly displayed larger within-subject fluctuations than GFAP and NFL. Nevertheless, a high correlation between UCHL1 and infarct volume was found 4.7 days after stroke onset, and similar to NFL, remained correlated with acute infarct volume after 90 days.

For these biomarkers to be clinically interpretable at an individual level, an increased understanding of factors contributing to their levels beyond time and infarct volume is necessary. There is a lack of precise knowledge of how these proteins are metabolized, eliminated, and affected by other clinical factors. Stroke subtype, etiology, location, and treatments may influence the magnitude and speed at which they enter the bloodstream. A multitude of comorbidities and clinical factors, such as concurrent cerebrovascular small vessel disease, renal failure, age, and BMI, may influence baseline levels and elimination rates [35, 36].

One way of overcoming some of these obstacles might be to analyze the rate of change rather than looking at absolute levels. For example, in one study, the rate of increase of GFAP and t-tau within the first 24 h after symptom onset outperformed neuroimaging in clinical outcome prediction (NIHSS at 24 h) after EVT, demonstrating a potential addition to clinical decision-making, i.e., to identify patients where reperfusion therapy might be futile [17]. In another study, a combination of absolute GFAP levels and rate of change ruled out hemorrhagic stroke with a negative predictive value of 98.4% [25].

For patients suffering from stroke, physical and cognitive function are naturally more important outcomes than the exact infarct volume. However, infarct volume is strongly associated with long-term outcomes, even though non-eloquent brain regions exist [21, 37]. In the current study, GFAP was the best biomarker for evaluating infarct volume, considering that this protein had the overall highest correlation to infarct volume, with the smallest confidence interval, throughout the whole week in this mixed ischemic stroke population with a wide range of infarct volumes. Beyond the first week, NFL and UCHL1 were the only biomarkers that, at 90 days, correlated with acute infarct volume, indicating that these biomarkers still hold information long after the ischemic event. Similar findings were reported for NFL in a previous study [18]. In patients with unknown symptom onset, serial sampling within a predefined time frame, possibly with a combination of two or more biomarkers, could be considered. Based on the result of the present study, a decrease in GFAP and an increase in NFL would indicate symptom onset more than 24 h ago, which is the current time limit for reperfusion treatment.

The significant variability of UCHL1 and t-tau poses a barrier to the interpretability of individual levels within the context of acute ischemic stroke. There is little benefit in measuring these proteins in addition to GFAP and NFL if the purpose is to determine infarct size through a single time point measurement. It is, therefore, encouraging that recent reports of a newly developed assay analyzing a CNS-specific tau isomer, *brain-derived* tau, have shown promising results, indicating that protein as an emerging biomarker in acute stroke management [38, 39].

This study has several limitations. Firstly, the number of patients was limited to 38, with only 33 patients having complete imaging data. This is explained by the negative impact of the COVID-19 pandemic on patient inclusion. Nevertheless, the final study population closely represents the average Swedish stroke population in terms of age, sex and stroke severity [40]. Secondly, information on comorbidities and treatments was collected through medical records, which might not be as accurate as data collected prospectively in a case report form. Thirdly, as less than half of the patients had both biomarker measurement at T_optimal_ ± 12 h and clinical outcome assessment at 90 days, no firm analysis of biomarker outcome prediction was possible. This was, however, not the main focus of this study. Fourthly, with only eleven patients having samples collected within the first 24 h of symptom onset, the study does not sufficiently capture biomarker changes during this period. Fifthly, infarct volume was measured between 48–72 h after symptom onset, a time point at which brain swelling may have slightly affected volume estimation. DWI-based studies show that swelling inflates apparent infarct volume by roughly one-third at ≈72 h (peak volume reached at 74 h; + 21% on day 2 and + 10% on day 3) [41]. However, since MRI were performed at the same time across individuals, this effect is expected to be consistent throughout the study.

In this high-resolution serial sampling of GFAP, NFL, t-tau, and UCHL1 in plasma over the first week after acute ischemic stroke, three distinct temporal patterns were observed. The most accurate assessments of infarct volume through these biomarkers occurred between four to six days after symptom onset. These findings may have implications for future studies and implementation in clinical practice.

## Supplementary Information

Below is the link to the electronic supplementary material.

ESM 1 (PDF 232 KB)

## Data Availability

Raw data are not publicly available to preserve individuals’ privacy under the European General Data Protection Regulation. A fully anonymized dataset supporting this article is available upon reasonable request to the corresponding author.

## Abbreviations

DWI: Diffusion-weighted images
eGFR: Estimated glomerular filtration rate
GFAP: Glial fibrillary acidic protein
IQR: Interquartile range
NFL: Neurofilament light
NIHSS: National Institutes of Health Stroke Scale
mRS: Modified Rankin scale
t-tau: Total tau
TIA: Transient ischemic attack
UCHL1: Ubiquitin carboxy-terminal hydrolase L1
