# Supplementary material for: Plasma Profiles of Neuroglial Injury Biomarkers after Ischemic Stroke
Source: Transl Stroke Res. 2025 Sep 3;16(6):2185–94. doi: 10.1007/s12975-025-01380-y (PMC12598674; doi:10.1007/s12975-025-01380-y)
Supplement: Supplementary file 1 — (PDF 232 KB) [file 12975_2025_1380_MOESM1_ESM.pdf]

Supplementary material

Plasma profiles of neuroglial injury biomarkers after ischemic stroke

Content

Figure S1-S4 **1-2**

Figure S1

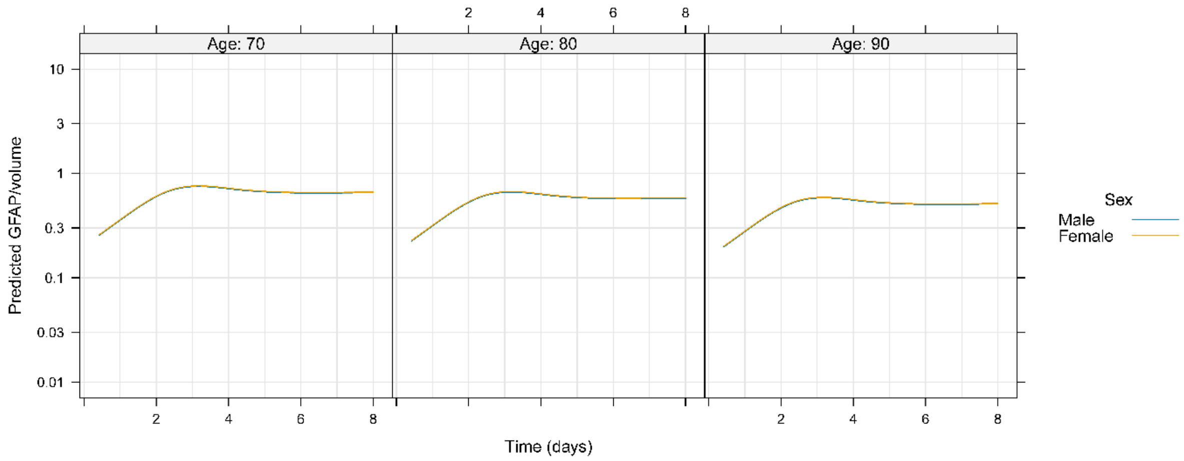

GFAP plasma profile adjusted for age and sex. The adjustments were done by fitting a linear mixed-effects model with the volume-normalized biomarker level as outcome, accounting for the correlation between measurements within the same subject. Time since onset, age and sex were included as fixed effects. The estimated average volume-normalized biomarker level over time was plotted using predictions from the model for both sexes (color) and three arbitrarily selected ages (panels).

Figure S2

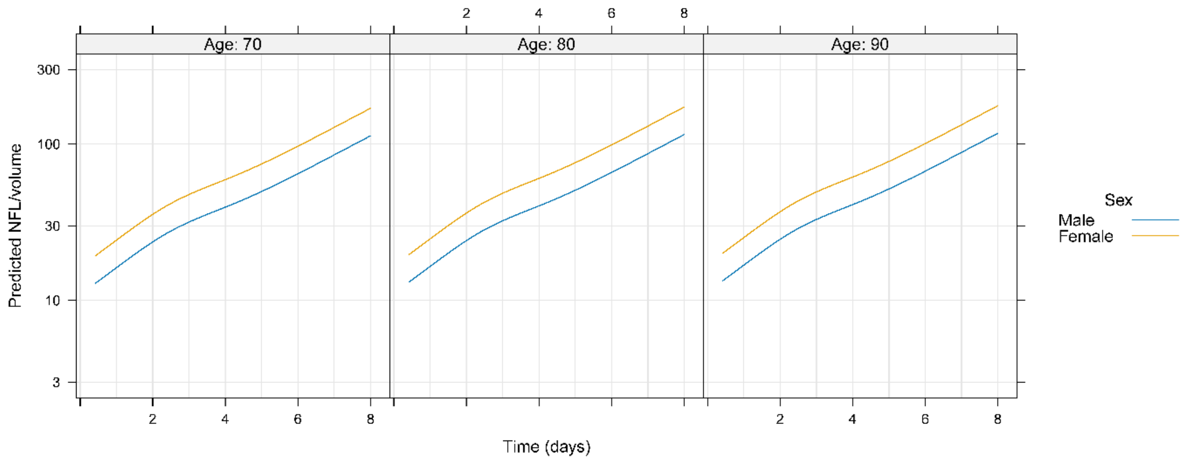

NFL plasma profile adjusted for age and sex. The adjustments were done by fitting a linear mixed-effects model with the volume-normalized biomarker level as outcome, accounting for the correlation between measurements within the same subject. Time since onset, age and sex were included as fixed effects. The estimated average volume-normalized biomarker level over time was plotted using predictions from the model for both sexes (color) and three arbitrarily selected ages (panels).

Figure S3

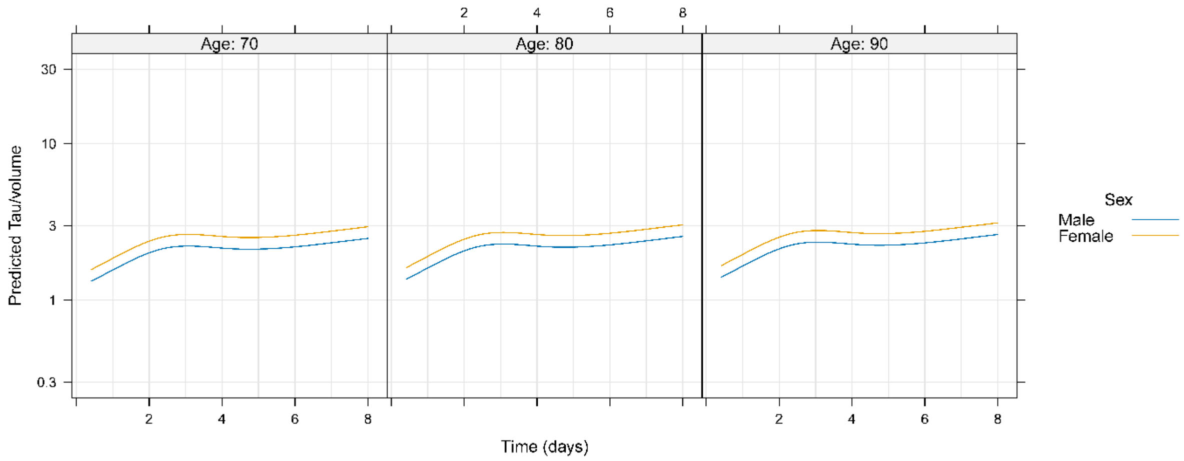

Tau plasma profile adjusted for age and sex. The adjustments were done by fitting a linear mixed-effects model with the volume-normalized biomarker level as outcome, accounting for the correlation between measurements within the same subject. Time since onset, age and sex were included as fixed effects. The estimated average volume-normalized biomarker level over time was plotted using predictions from the model for both sexes (color) and three arbitrarily selected ages (panels).

Figure S4

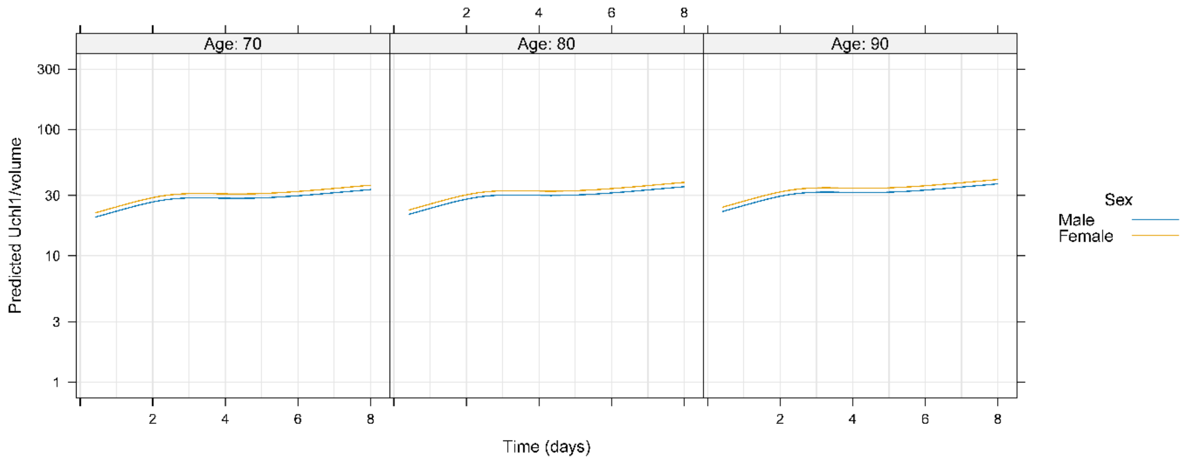

UCHL1 plasma profile adjusted for age and sex. The adjustments were done by fitting a linear mixed-effects model with the volume-normalized biomarker level as outcome, accounting for the correlation between measurements within the same subject. Time since onset, age and sex were included as fixed effects. The estimated average volume-normalized biomarker level over time was plotted using predictions from the model for both sexes (color) and three arbitrarily selected ages (panels).
